# Optimization of Antifungal Properties of Hop Cone Carbon Dioxide Extracts Based on Response Surface Methodology

**DOI:** 10.3390/molecules29112554

**Published:** 2024-05-29

**Authors:** Katarzyna Tyśkiewicz, Renata Tyśkiewicz, Marcin Konkol, Marcin Gruba, Rafał Kowalski

**Affiliations:** 1 Supercritical Extraction Research Group, Łukasiewicz Research Network—New Chemical Syntheses Institute, Al. Tysiąclecia Państwa Polskiego 13A, 24-110 Puławy, Poland; marcin.konkol@ins.lukasiewicz.gov.pl (M.K.) marcin.gruba@ins.lukasiewicz.gov.pl (M.G.); rafal.kowalski@ins.lukasiewicz.gov.pl (R.K.); 2 Analytical Laboratory, Łukasiewicz Research Network—New Chemical Syntheses Institute, Al. Tysiąclecia Państwa Polskiego 13A, 24-110 Puławy, Poland; renata.tyskiewicz@ins.lukasiewicz.gov.pl

**Keywords:** growth inhibition, hop cones, human protection, optimization, phytopathogens, response surface methodology

## Abstract

Response surface methodology (RSM) was employed to optimize the process parameters of the supercritical carbon dioxide extraction of hop cones in terms of their antifungal properties against *Fusarium culmorum* and *Aspergillus niger*. The effects of temperature (40–50 °C), pressure (200–300 bar), and CO_2_ consumption (25–75 kgCO_2_/kg) on the extraction yield, content of α- and β-acids, as well as pathogens’ growth inhibition were investigated. Both pressure and CO_2_ consumption had a significant effect on antifungal properties. It was observed that the best results for antifungal properties were obtained when hop cones were extracted with pure carbon dioxide at the temperature of 50 °C, under the pressure of 300 bar with CO_2_ consumption at the level of 75 kgCO_2_/kg of feed for extraction. The highest antifungal properties of hop cone supercritical carbon dioxide extracts were analyzed as 100% for *Fusarium culmorum* and 68% for *Aspergillus niger*, calculated as the growth inhibition of tested pathogens. The aim of the study was to determine the optimum values of extraction parameters to achieve the maximum response and enable us to investigate the interaction of these parameters on the antifungal properties of hop cone extracts.

## 1. Introduction

The worldwide beer market reached 1.89 billion hectoliters in 2023, resulting in a higher production of 1.3% as compared to 2022 [1]. Recently, the hop extract market was estimated in 2024 at the value of EUR 1.58 billion to further increase by 30% in the next years [2]. The biggest players in hop market are the USA and Germany. Second in line are European countries (Poland, Czech Republic, and Slovenia), but also England, China, Australia, and New Zealand [3]. On the other hand, a great number of studies have been performed on hop cones throughout the years. Only in the last decade, scientists published over 20,500 papers related to the keyword *Humulus lupulus* and over 190,500 papers related to the keyword hop/hops as based on various databases (1300 and 70,500 based on ScienceDirect; 1300 and 52,200 based on Web of Science; 800 and 2000 based on PubMed; 16,000 and 28,000 based on Google Scholar; and 1100 and 37,800 based on Scopus).

*Humulus lupulus*, commonly known as hops, is a species of flowering plant in the *Cannabaceae* family. It is native to Europe, Asia, and North America, and is primarily known for its use in brewing beer [4]. The plant is a vigorous, climbing vine with rough stems and serrated leaves arranged oppositely along the stem. Hops are dioecious, meaning there are separate male and female plants. The female plants produce cone-like structures called strobiles, which are used in brewing to impart bitterness, flavor, and aroma to beer. These cones contain lupulin glands, which contain essential oils and acids responsible for the characteristic bitterness and aroma of hops [5].

Apart from the most common compounds found in hop cones belonging to bitter acids (α- and β-acids) [3], there are at least several other bioactive compounds (essential oils and polyphenols) that make hop cones a feedstock with a broad range of microbiological properties [3,5,6]. Among various properties, hop cones contain compounds, such as prenylated flavonoids, which have been shown to possess sedative properties [7]. Certain compounds found in hops, such as phytoestrogens, have been investigated for their potential in hormone regulation. These compounds may have implications for conditions such as menopausal symptoms [8]. Moreover, hops contain various antioxidants, including polyphenols, which may help combat oxidative stress and reduce the risk of chronic diseases associated with oxidative damage [9] as well as possessing digestive-stimulant properties and helping alleviate gastrointestinal discomfort [10]. They contain compounds such as humulene and myrcene, which are believed to have relaxing effects [11,12,13]. There are at least several bioactive compound extraction methods offering various possibilities, with supercritical fluid extraction (SFE) as one of the green methods [14]. It is a method used to extract bioactive compounds from natural materials, such as plants, herbs, and botanicals. In the case of hop cones (*Humulus lupulus*), SFE has been utilized to extract essential oils, resins, and other compounds that contribute to the flavor, aroma, and bitterness in beer production. SFE involves using supercritical fluids, typically carbon dioxide (CO_2_) at specific temperature (above 31.1 °C) and pressure (above 73.8 bar) conditions, to extract target compounds from the raw material. In the supercritical state, CO_2_ exhibits both liquid-like and gas-like properties, allowing it to penetrate the plant material effectively and dissolve the desired compounds [15]. As far as different extraction yields may be obtained for different hop varieties [16], different extraction parameters may influence the chemical composition as well as antifungal properties of extracts [17]. Extracts from hops have demonstrated antimicrobial activity against various pathogens, suggesting potential applications in combating bacterial and fungal infections [18]. For instance, soft resins were tested against *Candida* spp., *Fusarium* spp., *Streptococcus* spp. and *Staphylococcus* spp., while oils were additionally tested on *Bacillus* spp. and *Escherichia coli* [18]. Olsovska et al. [18] also performed interesting research on the effects of hop cone polyphenols on herpes and influenza viruses. However, no study focused on choosing extraction parameters guaranteeing a relatively high pathogen-inhibition percentage. The optimization of any extraction process is a crucial aspect, especially when the total cost of manufacturing (COM) is considered that may affect the profitability of the final product [19]. In the case of the SFE method, there are at least several factors that affect the final yield and chemical composition of obtained extracts, including temperature, pressure, time, CO_2_ flow rate, and consumption. The most common method of parameter optimization is the response surface methodology (RSM), with the aim to understand the influence of the main chosen parameters on the SFE efficiency and measuring the optimal values for those factors [20].

Our study provides a new insight into the optimization of SFE parameters on the antifungal properties of scCO_2_ hop cone extracts against *Fusarium culmorum* and *Aspergillus niger* in order to obtain extracts with high antifungal properties. To date, no scientific paper has reported SFE optimization with the response surface methodology (RSM) that can test a broad range of extraction parameters on phytopathogen inhibition by scCO_2_ extract from hop cones. Taking into account the extraction of hops on a production scale, the optimization of parameters can significantly affect the costs of such a process [21].

## 2. Results and Discussion

### 2.1. Hop Cone scCO_2_ Extraction

Supercritical fluid extraction was applied for hop cone extraction when the two-step extraction was introduced by Pfaf-Šovljanski et al. [22]. In the first step, extraction was conducted at the temperature of 40 °C, under the pressure of 150 bar for 2.5 h, followed by an increase in the pressure to 300 bar. The aim of the study was to increase the extraction efficiency of Magnum hops to the total of 20.89 wt% [22]. In further studies, Zeković et al. [23] applied a two-step extraction (50 °C/150 bar; 50 °C/300 bar) to other hop cone varieties, with the extraction yields of 8.64, 11.40, 10.63, and 9.44 wt% for Hallertau Tradition, Spalt Selekt, Aroma, and K-62, respectively. Nagybákay et al. [24] used the central composite design (CCD) for the optimization of the extraction parameters of *Ella* hops in terms of extraction yield. The best results (26.32 wt%) were obtained when the extraction was performed at the temperature of 43 °C and under the pressure of 370 bar for 80 min. Experimental parameters employed by Kupski et al. [25] were not as effective for hop cones as in our study. The researchers obtained the extraction yield of Hallertau hops in the range of 1.2–7.1 wt%, with the use of the following parameters: 35 °C, 100 and 200 bar, 45 °C and 150 bar, as well as 55 °C, 100 and 200 bar [26]. The supercritical fluid extraction of Marynka hop cones resulted in an extraction yield in the range of 9.21–16.23 wt% (Table 1). The lowest extraction yield was obtained for the extraction temperature of 40 °C, extraction pressure of 250 bar, and CO_2_ consumption of 25 kgCO_2_/kg. With the increase in the SFE parameters to 50 °C, 300 bar, and 75 kgCO_2_/kg, an increase in the extraction yield was observed to 16.23 wt%.

**Figure 1:**
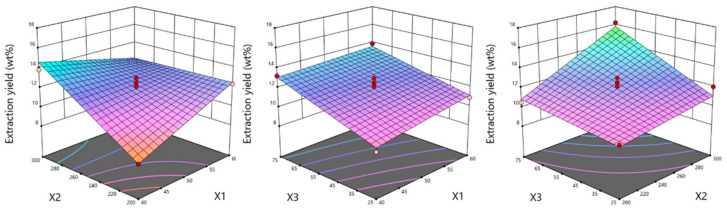
The influences of temperature (X_1_) and pressure (X_2_), temperature (X_1_) and CO_2_ consumption (X_3_), as well as pressure (X_2_) and CO_2_ consumption (X_3_) on Marynka hop cones’ extraction yield (wt%).

Different models were statistically assessed to find the proper model to evaluate the influence of extraction parameters on hop cones’ extraction yield. On the basis of the Box–Behnken methodology, a good fit was found for the 2FI (two-factor interaction) model (R^2^ = 0.9097). The *p*-value was much lower than 0.05, which indicated that the model as well as particular model terms (pressure, CO_2_ consumption, temperature × pressure, and pressure × CO_2_ consumption) were significant. With the increase in the extraction pressure from 200 to 300 bar at a constant temperature (50 °C), the extraction yield also increased from 9.88 to 16.23 wt%. The opposite correlation was observed for the increase in temperature from 40 to 60 °C, which decreased the extraction yield from 12.83 to 11.34 wt% (Figure 1).

### 2.2. Bioactive Compounds in Hop Cone scCO_2_ Extracts

The results of 17 runs are shown in Table 2. The sum of α- and β-acids was analyzed to be in the range of 48.59–57.25 wt%. The lowest content was obtained in Exp. 7 (60 °C, 250 bar, and 75 kgCO_2_/kg), while the highest α- and β-acid contents were characterized in Exp. 1 (40 °C, 300 bar, and 50 kgCO_2_/kg). Rój et al. [26] obtained 60.5 wt% bitter acids in Marynka extract at the temperature of 50 °C and 300 bar.

Supercritical carbon dioxide extract from Exp. 1 is characterized by the highest contents of cohumulone (8.65 wt%) and N+adhumulone (28.33 wt%), while the highest contents of colupulone (10.81 wt%) and N+adlupulone (10.35 wt%) were observed in Exp. 15 and Exp. 3, respectively. The sum of β-acids was the highest in the extract from Exp. 15 (21.09 wt%). Similar to the extraction yield, the increase in the pressure from 200 to 300 bar caused the increase in the sum of bitter acids from 56.87 to 57.12 wt%. In the case of the extraction temperature, the content of bitter acids decreased from 55.92 to 53.76 wt% with the increase in the temperature from 40 to 60 °C.

The highest content of α-acids (41.0 wt%) among different hop cone varieties was analyzed in Magnum, while the lowest (9.8–9.9 wt%) in Spalt Selekt and K-62 extracts [23]. The ratio of α- to β-acids obtained in our extracts was 1.7–1.9/1 and was similar to the ratio of bitter acids in the study by Del Valle et al. [27], who focused on Nugget, Osorno, and Eizalde Lake hops varieties.

**Figure 2:**
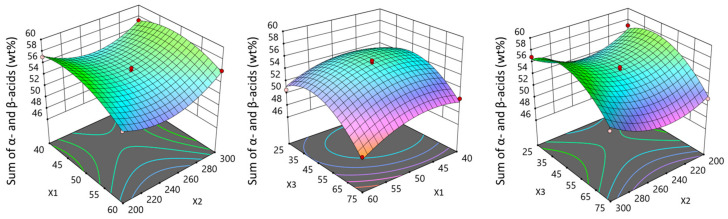
The influence of temperature (X_1_) and pressure (X_2_), temperature (X_1_) and CO_2_ consumption (X_3_), as well as pressure (X_2_) and CO_2_ consumption (X_3_) on Marynka hop cones’ α- and β-acid contents (wt%).

**Figure 3:**
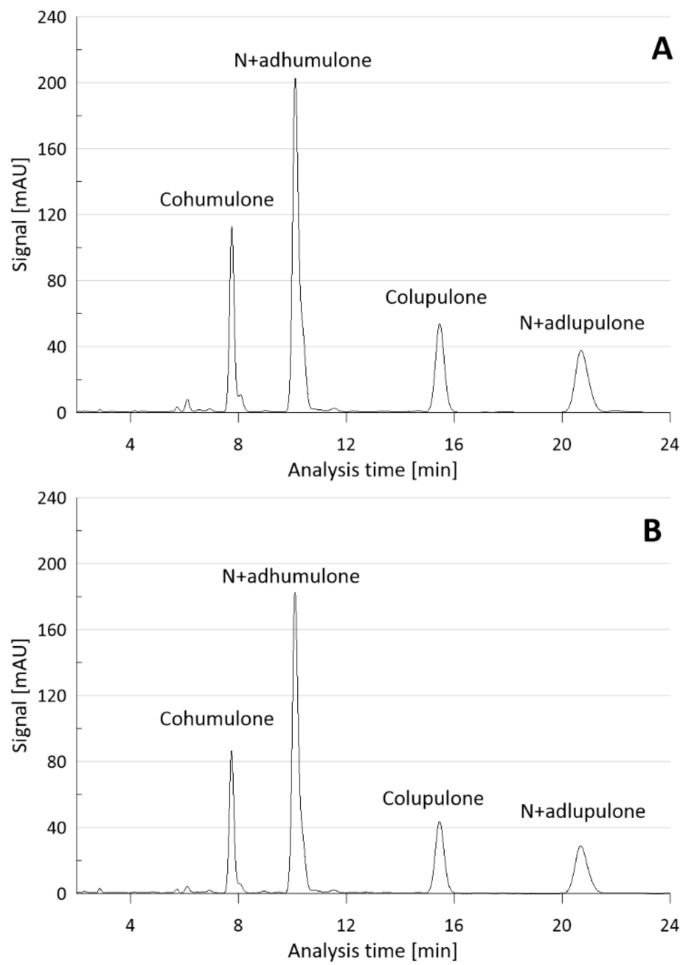
HPLC chromatogram of α- and β-acids ((**A**)—α- and β-acid standards; (**B**)—α- and β-acids identified in Exp. 16).

Analysis of variance (ANOVA) showed that the sum of α- and β-acids was most suitably described with a full quadratic model, resulting in 0.9704, 0.9323, and 0.7333, respectively, for R^2^, Adjusted R^2^, and Predicted R^2^. A good fit for the model did not require a reduction in model terms, which in some cases may improve R^2^. Among nine model terms resulting from the quadratic model, temperature, CO_2_ consumption, pressure × pressure, and CO_2_ consumption × CO_2_ consumption were significant for the sum of α- and β-acids (*p*-value less than 0.05) (Figure 2). The chromatogram of α- and β-acids is presented in Figure 3.

The highest content of separated pigments was detected in extracts obtained from hop cones, where the contents of chlorophyll A, chlorophyll B, and carotenoids were 178.92 (Exp. 11), 155.36 (Exp. 16), and 167.86 mg/kg (Exp. 11), respectively. The lowest content of chlorophyll A (38.41 mg/kg) was detected in the extract from Exp. 10; the lowest contents of chlorophyll B (49.22 mg/kg) and carotenoids (25.86 mg/kg) were detected in extracts from Exp. 3 (Table 3). A lower content of chlorophyll A was analyzed at the level of 146.13 mg/kg of extract and almost 1.5-fold-more carotenoids were detected in Ella hops as compared to Exp. 11 [24]. The highest extraction yield (16.23%) resulted in the highest content of pigments (481.81 mg/kg) in Marynka hop cone scCO_2_ extracts.

### 2.3. Antifungal Properties

Supercritical fluid extraction parameters were previously optimized in terms of flavonoids [28] and essential oil [29], apart from extraction yield [23,30]. However, none of the research papers provide insights into influence of the optimization of extraction parameters on the antifungal properties of hop cone extracts. The literature data indicate the antimicrobial properties of *H. lupulus* extracts against various pathogens. For instance, Schoss et al. [31] showed high antifungal properties of common hops’ scCO_2_ extract against *A. alternata*, *E. nigrum*, *F. oxysporum*, and *B. cinerea*, with their growth inhibition at the levels of 72.32%, 81.18%, 67.10%, and 76.87%, respectively. There are at least several scientific papers reporting the antifungal properties of plant extracts obtained with scCO_2_. For instance, cedar oils were tested against *Gloeophyllum trabeum* and *Trametes versicolor* [32]. Our previous study evaluated the influence of the scCO_2_ extracts of *Fucus vesiculosus* against *Fusarium culmorum* and *Fusarium oxysporum* [33]. Bai et al. [34] used the root extract of *Stellera chamaejasme* to fight against *Monilinia fructicola*.

**Figure 4:**
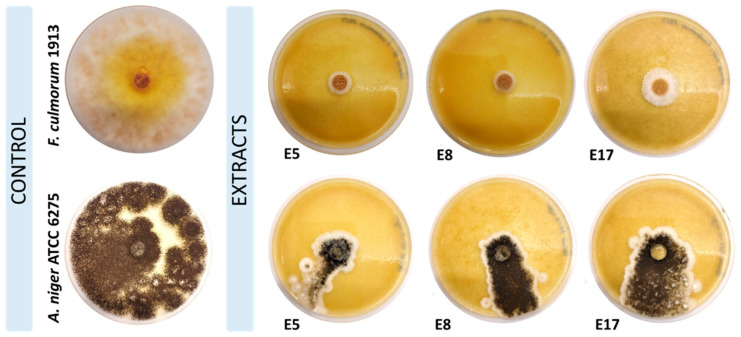
The influence of hop cone scCO_2_ extracts (E5—Exp. 5; E8—Exp. 8; E17—Exp. 17) on *F. culmorum* 1913 and *A. niger* ATCC 6275 growth inhibition (%).

The best antifungal properties (100% of growth inhibition) of hop cone scCO_2_ extracts against phytopathogenic *F. culmorum* were observed in Exp. 8 (50 °C, 200 bar, and 75 kgCO_2_/kg). The mentioned pathogen was the most resistant to extract from Exp. 11, which was able to inhibit the growth of *F. culmorum* only by 65%. A similar effect (67.10%) was achieved for *F. oxysporum* in the studies by Schoss et al. [31]. Several extracts (Exp. 3, 4, 5, 7, and 14) were characterized by the same antifungal properties in the range of 81–84%. As it was observed, hop cone extracts obtained with scCO_2_ differed in terms of antifungal properties among tested pathogens, with a lower effect for *A. niger* (range of 39–68% of growth inhibition) in a comparison with *F. culmorum* (Table 4, Figure 4). The minimal fungicidal concentration of phenolic compound-based extract from hop seeds to inhibit *A. niger* ATCC 6275 growth was determined at the level of 0.60 mg/mL [35]. Water-based extract of milled hop cones inhibited the growth of *A. niger* DPPMAF3, calculated as approx. 22% after six days of incubation on PDA medium at 25 °C [36]. Hop cone extracts obtained with organic solvents (ethyl acetate, acetone, and methanol) were characterized by worse antifungal properties against *F. culmorum* AM10 (53.1–55.9%) in regard to the results presented in this study (65–100%) [37]. Hop cone extracts from Exp. 8 and Exp. 10 were characterized by the lowest content of pigments (142.41 and 114.18 mg/kg, respectively). As determined by Avalos and Carmen Limón [38] and Naz et al. [39], the lower the content of carotenoids, the higher the inhibition percentage of fungi growth, as carotenoids having antioxidant properties may enhance the survival of phytopathogens in a natural environment and protect them against severe conditions (influences of stress and light).

Optimization methods have been broadly used in microbiological studies in terms of different factors. For instance, the response surface methodology was applied to optimize culture medium components (composed of peptone, sucrose, and yeast) for biofungicide production by *Bacillus amyloliquefaciens* [40]. Another optimization method (2^3^ factorial design) was used to optimize the antifungal properties of polyurethane (PUR) and silver nanoparticles (AgNPs) composites against the fungus *Trichophyton rubrum* [41]. El-Housseiny et al. [42] utilized the response surface methodology to test the effects of different temperatures, pH, and inoculum sizes on the antifungal properties of *B. subtillis* subsp. Spizizenii culture against *Candida albicans*.

**Figure 5:**
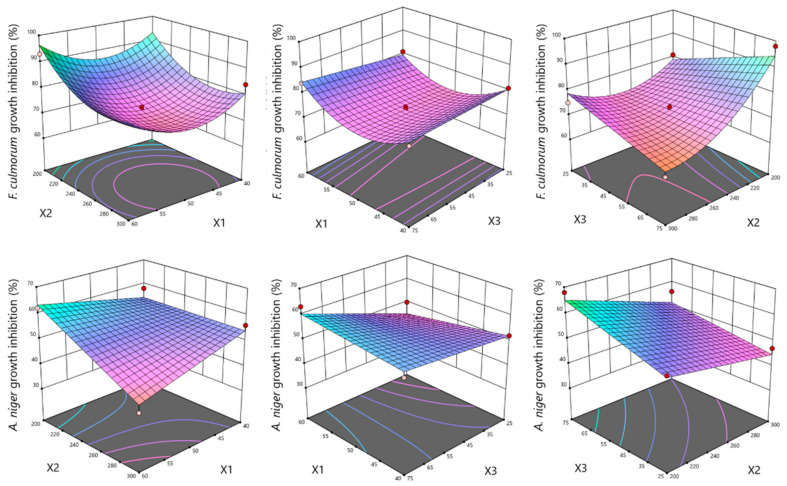
The influence of temperature (X_1_) and pressure (X_2_), temperature (X_1_) and CO_2_ consumption (X_3_), as well as pressure (X_2_) and CO_2_ consumption (X_3_) on *F. culmorum* 1913 and *A. niger* ATCC 6275 growth inhibition (%).

The ANOVA analysis for the antifungal properties of hop cone scCO_2_ extracts against *F. culmorum* and *A. niger* indicated the reduced quadratic model and 2FI model to be statistically significant in terms of the studied parameters, respectively. As far as *F. culmorum* is concerned, the fit for the model was 0.9339 (R^2^), with model terms such as pressure (X_2_), pressure × CO_2_ consumption (X_2_X_3_), as well as temperature × temperature (X_1_^2^) and pressure × pressure (X_2_^2^) being statistically significant (*p*-values less than 0.05). The optimal conditions for antifungal properties against *F. culmorum* were 58 °C, 201 bar, and 69 kgCO_2_/kg. The influence of hop cone extracts on *A. niger* growth inhibition were pressure (X_1_), CO_2_ consumption (X_3_), as well as temperature × pressure (X_1_X_2_) (Figure 5). The results show a good fit to the proposed model and the optimal conditions obtained (210 bar, 60 °C, and CO_2_ consumption of 70 kgCO_2_/kg) are within the experimental range. The predicted values agreed with the experimental ones, thus indicating the suitability of the RSM model for the optimization of the extraction conditions being investigated.

With the increase in pressure from 200 to 300 bar at the constant temperature of 40 °C, the antifungal properties of hops extract decreased from 91 to 81% (Exp. 15 and Exp. 1) and from 58 to 56% (Exp. 15 and Exp. 1), respectively, for *F. culmorum* and *A. niger*. No influence of temperature increase from 40 to 60 °C was observed in Exp. 5 and Exp. 7 on *F. culmorum* and *A. niger* growth inhibition. At a constant pressure (250 bar) and the same CO_2_ consumption of 25 kgCO_2_/kg, the growth inhibition of the studied pathogens were analyzed at the level of 81% for *F. culmorum* and 50–52% for *A. niger*. Response surface methodology was used for antibacterial and antifungal assays for organic extracts [43,44], yet a small number of publications are devoted to supercritical carbon dioxide extracts [45]. Pereira et al. [45] optimized the SFE parameters of myrtle (*Myrtus communis* L.) with the response surface methodology in order to obtain one extract with higher antibacterial activity against various bacteria (*S. aureus*, *B. subtilis*, and *S. epidermidis*) than antibiotics tested (vancomycin and norfloxacin). Response surface methodology was also a good choice for the determination of a strong correlation between organic solvents extracts of *Areca nut* and *Punica granatum* against *E. coli*, *S. aureus*, and *Salmonella* [43]. Inhibitory zone diameter (mm) was a criterion for extraction parameter optimization by the RSM method for *Ficus hirta* fruit ethanolic extracts against *Penicillum italicum* and *Penicillum digitatum* [44].

The equations for dependent variables were obtained as follows:

Extr. yield = 12.06 +0.47X_1_ + 1.62X_2_ + 1.48X_3_ − 1.60X_1_X_2_ − 0.24X_1_X_3_ + 0.91X_2_X_3_ − 0.51X_1_^2^ (1)

Sum of α- and β-acids = 55.28 − 1.19X_1_ + 0.51X_2_ − 1.90X_3_ + 0.52X_1_X_2_ − 0.45X_1_X_3_ + 0.45X_2_X_3_ − 1.88X_1_^2^ + 2.67X_2_^2^ − 3.40X_3_^2^ (2)

Antifungal properties (*F. culmorum* 1913) = 72.79 + 1.00X_1_ − 6.87X_2_ + 0.87X_3_ + 1.50X_1_X_3_ − 6.75X_2_X_3_ + 8.22X_1_^2^ + 6.97X_2_^2^ (3)

Antifungal properties (*A. niger* ATCC 6275) = 53.29 − 0.75X^1^ − 5.38X^2^ + 4.88X^3^ − 5.25X^1^X^2^ + 2.75X^1^X^3^ − 1.50X^2^X^3^ (4)

## 3. Materials and Methods

### 3.1. Materials

#### 3.1.1. Plant Collection and Sample Preparation

Dried Marynka hop cones (*Humulus lupulus*) were used as an experimental raw material in this study. The geographic origin of crops was the Lublin province, Poland. Cones with a moisture content of 9.3% were milled on a Retsch SM100 mill (Katowice, Poland) using a sieve with a mesh size of 1.5 mm. The average particle size was 0.9 mm. The produced material was subjected to extraction with supercritical carbon dioxide using a laboratory scale plant.

#### 3.1.2. Fungal Culture Collection and Culture

Fungal strain *F. culmorum* 1913 was obtained from the fungi collection of the Plant Diseases Clinic and Bank of Pathogens of the Institute of Plant Protection—NRI in Poznań, Poland. *A. niger* ATCC 6275 (American Type Culture Collection) was purchased from Microbiologics Company, St Cloud, MN, USA. PDA (Potato Dextrose Agar) medium was purchased from BioMaxima Company, Lublin, Poland. *F. culmorum* 1913 was isolated from corn (*Zea mays*) in Winna Góra (Poland), whereas the isolation source of *A. niger* ATCC 6275 was leather.

#### 3.1.3. Chemicals and Software

Carbon dioxide (99.9%, *v*/*v*), which was used as the mobile phase in SFE, was stored in a CO_2_ installation tank. Methanol, diethyl ether, and phosphoric acid were purchased from Witko (Łódź, Poland). The standard of bitter acids (ICE-4) consisting of 42.58 and 26.54 wt%, respectively, of α- and β-acids was supplied from Labor Veritas AG, Zürich, Switzerland.

### 3.2. Methods

#### 3.2.1. Supercritical Fluid Extraction (SFE)

The dynamic SFE process was performed in the Łukasiewicz Research Network–New Chemical Syntheses Institute (Puławy, Poland) in accordance with the design of experiments (DOEs) based on the Box–Behnken methodology. SFE was performed in a laboratory plant equipped with a 1 dm^3^ extractor (SITEC, Zurich, Switzerland). Maximum operating temperature was up to 200 °C, while pressure was up to 500 bar. The complete design included fifteen experiments at different conditions with the use of three center points. The independent variables at three levels were chosen as temperature (40 (−1); 50 (0); 60 (1)), pressure (200 (−1); 250 (0); 300 (1)), and CO_2_ consumption (25 (−1); 50 (0); 75 (1)). The CO_2_ flow (10 kg/h) was constant for all experiments. In order to evaluate the influence of these factors, the extraction yield (dependent variable 1), the contents of α- and β-acids (dependent variable 2), as well as antifungal properties (dependent variable 3) were determined and used to optimize the extraction conditions. The statistical analysis of the results was performed with Design Expert 13.0 software (Stat-Ease, Minneapolis, MN, USA).

#### 3.2.2. High-Performance Liquid Chromatography (HPLC)

The hop cone scCO_2_ extracts were analyzed using Agilent Technologies 1260 Infinity II liquid chromatograph equipped with a UV detector in terms of α- and β-acid contents. The separation was carried out using an Agilent Zorbax Eclipse Plus C18 column (Santa Clara, CA, USA) (4.6 mm × 100 mm × 3.5 μm) at the temperature of 40 °C. The mobile phase used a mixture of methanol, water, and orthophosphoric acid in a ratio of 775:225:9 at the flow rate of 1 mL/min in an isocratic mode. The injected sample volume was 10 μL and the detector was set at 314 nm.

The ICE-4 standard (approx. 0.5 g) was diluted in 60 mL of methanol followed by transferring the solution to a 100 mL flask and filling it up with methanol to beaker. The solution was diluted 5 times and filtered through a 0.45 μm syringe filter, and then subjected to chromatographic analysis. Similar to the standard, the real samples were prepared by weighing an amount of approx. 0.5 g. Calculations of the content of α- and β-acids in the tested sample are carried out according to the following formula.

(5) $$X_{n=\frac{m_{std}}{m}\;\times \;\frac{A}{A_{std}}\;\times \;C_{std}}$$

where *X_n_*—the content of analyzed compound (wt%); *m_std_*—mass of standard (g); *m*—mass of tested samples (g); *A*—peak area of the determined sample component; *A_std_*—peak area of the determined standard component; and *C_std_*—wt% content of the determined ingredient in the standard (according to the certificate).

#### 3.2.3. Pigment Content

The contents of chlorophyll A, chlorophyll B, and carotenoids were determined spectrophotometrically on the basis of the previously described methodology [46,47]. Simply, all samples of hop cone extracts were mixed with diethyl ether and subjected to ultrasound for 30 min. The mixture was then centrifuged for 20 min at 7000 rpm. The supernatant was separated and the samples were analyzed at absorbances of 662 nm (chlorophyll A), 646 nm (chlorophyll B), and 470 nm (carotenoids). The amounts of these pigments were calculated according to the following formulas:

C_A_ = 10.05A_662_ − 0.766A_646_ (6)

C_B_ = 16.37A_646_ − 3.140A_662_ (7)

C_CAR_ = 1000A_470_ − 1.280C_A_ − (56.7C_B_)/230 (8)

where C_A_ is chlorophyll A, C_B_ is chlorophyll B, and C_CAR_ is carotenoids.

#### 3.2.4. Antifungal Properties

The crude extracts (1 mL) were transferred separately to 20 mL of dissolved PDA medium, cooled to 45 °C and mixed thoroughly. The mixtures were then transferred to a Petri dish (90 mm diameter) and allowed to solidify. The final and tested concentration of extracts was 5 wt%. Agar discs (9.0 mm diameter) overgrown with *Fusarium culmorum* 1913 and *Aspergillus niger* ATCC 6275 mycelium were taken from the initial cultures (grown on PDA medium for 5 days at 27 °C) and transferred to the center of the prepared medium (PDA + extract). Additionally, a negative control (PDA + fungus) was included. All plates were then incubated at 27 °C for 5 days. The effect of extracts was determined by measuring the diameter of the colonies and expressed as the percentage inhibition of mycelial growth compared to the negative control.

## 4. Conclusions

The present study showed for the first time the effect of SFE parameters on the antifungal properties of hop cone extracts against *Fusarium culmorum* and *Aspergillus niger* growth inhibition optimized by the response surface methodology. The reference *F. culmorum* 1913 and *A. niger* ATCC 6275 species were used for the study. The results reveal *F. culmorum* 1913 growth inhibition in the range of 65–100%, as well as hop cone extracts inhibiting 39–68% of *A. niger* ATCC 6275 growth. Different contents of bioactive compounds (bitter acids and carotenoids) were attributed to different antifungal properties. The best results in terms of extraction yield were obtained for hop cone extraction (extraction yield = 16.23 wt.%) at the temperature of 50 °C, under the pressure of 300 bar with 75 kgCO_2_/kg of feed for extraction. These SFE conditions resulted in *F. culmorum* 1913 and *A. niger* ATCC 6275 growth inhibitions of 65 and 53%, respectively. Within the criterion of the highest antifungal properties, the best extract against *F. culmorum* and *A. niger* was obtained at 50 °C, 200 bar, and 50 kgCO_2_/kg. The presented results are expected to contribute to the valorization of hop cone extract as an agent with antifungal properties. This study opens new possibilities to fight against serious pathogens.

## Figures and Tables

**Table 1:** Experimental datasets for extraction yield (wt.%) of hop cone scCO_2_ extracts using the CCD design.

Exp.	Temperature, °C		Pressure, Bar		CO_2_ Consumption, kgCO_2_/kg		Extraction Yield, wt%
	U	C	U	C	U	C	
1	40	−1	300	1	50	0	12.83
2	50	0	250	0	50	0	12.09
3	50	0	200	−1	25	−1	9.88
4	60	1	250	0	25	−1	10.87
5	40	−1	250	0	25	−1	9.21
6	40	−1	250	0	75	1	13.21
7	60	1	250	0	75	1	13.89
8	50	0	200	−1	75	1	10.47
9	50	0	250	0	50	0	13.47
10	60	1	200	−1	50	0	12.28
11	50	0	300	1	75	1	16.23
12	50	0	250	0	50	0	11.19
13	50	0	250	0	50	0	10.31
14	60	1	300	1	50	0	11.34
15	40	−1	200	−1	50	0	10.08
16	50	0	300	1	25	−1	12.09
17	50	0	250	0	50	0	12.94

C—coded model terms; U—uncoded model terms.

**Table 2:** The content of α- and β-acids (wt%) in hop cone scCO_2_ extracts.

Exp.	Cohumulone	N + Adhumulone	Colupulone	N + Adlupulone	Sum of α-Acids	Sum of β-Acids	Sum of Bitter Acids
	wt%						
1	8.65 ± 0.23	28.33 ± 0.11	10.39 ± 0.11	9.88 ± 0.05	36.98 ± 0.03	20.27 ± 0.04	57.25 ± 0.03
2	8.46 ± 0.21	27.79 ± 0.08	10.12 ± 0.05	9.55 ± 0.03	36.25 ± 0.04	19.67 ± 0.03	55.92 ± 0.09
3	8.58 ± 0.15	27.36 ± 0.08	10.73 ± 0.03	10.35 ± 0.02	35.94 ± 0.09	21.08 ± 0.11	57.02 ± 0.10
4	7.26 ± 0.11	23.75 ± 0.12	9.01 ± 0.05	8.57 ± 0.04	31.01 ± 0.00	17.58 ± 0.03	48.59 ± 0.23
5	7.68 ± 0.05	25.13 ± 0.02	9.90 ± 0.09	9.53 ± 0.03	32.81 ± 0.04	19.43 ± 0.04	52.24 ± 0.04
6	8.38 ± 0.09	27.22 ± 0.01	10.08 ± 0.12	9.62 ± 0.01	35.60 ± 0.10	19.70 ± 0.06	55.30 ± 0.05
7	7.42 ± 0.12	24.24 ± 0.01	8.84 ± 0.20	8.37 ± 0.05	31.66 ± 0.06	17.21 ± 0.06	48.87 ± 0.05
8	7.62 ± 0.02	24.95 ± 0.02	9.58 ± 0.05	9.15 ± 0.05	32.57 ± 0.08	18.73 ± 0.04	51.30 ± 0.12
9	8.25 ± 0.03	26.93 ± 0.04	9.97 ± 0.00	9.59 ± 0.06	35.18 ± 0.01	19.56 ± 0.03	54.74 ± 0.11
10	8.02 ± 0.12	26.04 ± 0.11	10.03 ± 0.01	9.67 ± 0.08	34.06 ± 0.01	19.70 ± 0.02	53.76 ± 0.10
11	8.21 ± 0.32	26.77 ± 0.12	9.32 ± 0.02	8.72 ± 0.01	34.98 ± 0.01	18.04 ± 0.02	53.02 ± 0.07
12	8.19 ± 0.11	26.37 ± 0.14	10.04 ± 0.13	9.64 ± 0.02	34.56 ± 0.02	19.68 ± 0.01	54.24 ± 0.08
13	8.38 ± 0.01	27.06 ± 0.32	10.24 ± 0.12	9.83 ± 0.02	35.44 ± 0.02	20.07 ± 0.12	55.51 ± 0.05
14	8.45 ± 0.12	27.34 ± 0.01	10.37 ± 0.00	9.91 ± 0.01	35.79 ± 0.05	20.28 ± 0.03	56.07 ± 0.03
15	8.50 ± 0.21	27.53 ± 0.03	10.81 ± 0.00	10.28 ± 0.02	36.03 ± 0.05	21.09 ± 0.02	57.12 ± 0.12
16	8.62 ± 0.05	28.04 ± 0.02	10.38 ± 0.11	9.83 ± 0.01	36.66 ± 0.02	20.21 ± 0.05	56.87 ± 0.10
17	8.38 ± 0.04	27.52 ± 0.11	10.27 ± 0.05	9.76 ± 0.03	35.90 ± 0.00	20.03 ± 0.05	55.93 ± 0.09

**Table 3:** The content of pigments (mg/kg of extract) in hop cone scCO_2_ extracts.

Exp.	Chlorophyll A, mg/kg	Chlorophyll B, mg/kg	Carotenoids, mg/kg
1	81.59 ± 0.00	77.21 ± 0.01	65.81 ± 4.66
2	85.48 ± 0.05	64.24 ± 0.00	57.26 ± 0.18
3	41.24 ± 0.05	49.22 ± 0.01	25.86 ± 0.35
4	60.45 ± 0.00	69.75 ± 0.02	56.87 ± 5.36
5	45.98 ± 0.00	64.22 ± 0.02	39.97 ± 6.45
6	78.67 ± 0.00	93.60 ± 0.01	94.65 ± 2.19
7	56.16 ± 0.04	61.90 ± 0.01	39.22 ± 0.46
8	43.10 ± 0.00	57.99 ± 0.00	41.32 ± 3.47
9	61.58 ± 0.06	64.29 ± 0.02	48.10 ± 0.19
10	38.41 ± 0.03	49.62 ± 0.00	26.15 ± 0.65
11	178.92 ± 0.05	135.03 ± 0.01	167.86 ± 0.38
12	53.90 ± 0.00	77.82 ± 0.01	52.88 ± 4.89
13	60.93 ± 0.05	75.89 ± 0.00	67.90 ± 0.29
14	55.04 ± 0.00	77.60 ± 0.02	59.74 ± 6.84
15	49.63 ± 0.02	67.35 ± 0.01	46.97 ± 0.11
16	129.94 ± 0.01	155.36 ± 0.02	153.79 ± 8.84
17	116.48 ± 0.04	105.10 ± 0.01	135.63 ± 0.23

**Table 4:** Antifungal properties of Marynka hop cone scCO_2_ extracts against *F. culmorum* 1913 and *A. niger* ATCC 6275 expressed as growth inhibition (%) with standard deviation (SD).

Exp.	SFE Parameters (Temperature, °C; Pressure, Bar; CO_2_ Consumption, kgCO_2_/kg)	*F. culmorum* 1913 Growth Inhibition, % ± SD	*A. niger* ATCC 6275 Growth Inhibition, % ± SD
1	40, 300, 50	84 ± 2	56 ± 2
2	50, 250, 50	73 ± 2	51 ± 3
3	50, 200, 25	81 ± 1	53 ± 3
4	60, 250, 25	81 ± 1	50 ± 2
5	40, 250, 25	81 ± 0	52 ± 3
6	40, 250, 75	78 ± 1	54 ± 2
7	60, 250, 75	84 ± 1	63 ± 2
8	50, 200, 75	100 ± 0	68 ± 2
9	50, 250, 50	72 ± 1	52 ± 3
10	60, 200, 50	93 ± 1	62 ± 1
11	50, 300, 75	65 ± 1	56 ± 4
12	50, 250, 50	74 ± 1	48 ± 3
13	50, 250, 50	73 ± 1	52 ± 3
14	60, 300, 50	84 ± 1	39 ± 3
15	40, 200, 50	91 ± 2	58 ± 1
16	50, 300, 25	75 ± 2	47 ± 1
17	50, 250, 50	72 ± 1	45 ± 0

## Data Availability

All data will be made available on request.

## References

[1] Raiser T. World Beer Market Achieves Growth in Challenging Times, BarthHass Report 2022/2023. https://www.barthhaas.com/fileadmin/user_upload/PR_world-beer-market_2023.pdf.

[2] Wood L. (2024). Hops Extracts Market Poised for Growth: Comprehensive Industry Analysis Reveals Key Trends and Forecast to 2031.

[3] Almaguer C., Schönberger C., Gastl M., Arendt E.K., Becker T. (2014). *Humulus lupulus*—A story that begs to be told. A review. J. Inst. Brew..

[4] Przybyś M., Skomra U. (2020). Hops as a source of biologically active compounds. Pol. J. Agron..

[5] Kowalska G., Bouchentouf S., Kowalski R., Wyrostek J., Pankiewicz U., Mazurek A., Sujka M., Włodarczyk-Stasiak M. (2022). The hop cones (*Humulus lupulus* L.): Chemical composition, antioxidant properties and molecular docking simulations. J. Herb. Med..

[6] Tyśkiewicz K., Gieysztor R., Konkol M., Szałas J., Rój E. (2018). Essential oil from *Humulus Lupulus* scCO_2_ extract by hydrodistillation and microwave-assisted hydrodistillation. Molecules.

[7] Franco L., Sánchez C., Bravo R., Rodríguez A.B., Barriga C., Romero E. (2012). The sedative effect of non-alcoholic beer in healthy female nurses. PLoS ONE.

[8] Miranda C.L., Stevens J.F., Helmrich A., Henderson M.C., Rodriguez R.J., Yang Y.H., Deinzer M.L., Barnes D.W. (1999). Antiproliferative and cytotoxic effects of prenylated flavonoids from hops (*Humulus lupulus*) in human cancer cell lines. Food Chem. Toxicol..

[9] Stevens J.F., Page J.E. (2004). Xanthohumol and related prenylflavonoids from hops and beer: To your good health!. Phytochemistry.

[10] Yamamoto Y., Gaynor J.J., Yu B. (2016). Green tea catechins, cholesterol consumption, and long-term exercise are associated with delayed onset of high-fat diet-induced obesity and metabolic syndrome in rats. J. Nutr..

[11] Hartsel J.A., Eades J., Hickory B., Makriyannis A. (2016). Cannabis sativa and hemp. Nutraceuticals.

[12] Rufino A.T., Ribeiro M., Sousa C., Judas F., Salgueiro L., Cavaleiro C., Mendes A.F. (2015). Evaluation of the anti-inflammatory, anti-catabolic and pro-anabolic effects of E.-caryophyllene, myrcene and limonene in a cell model of osteoarthritis. Eur. J. Pharmacol..

[13] Fernandes E.S., Passos G.F., Medeiros R., da Cunha F.M., Ferreira J., Campos M.M., Pianowski L.F., Calixto J.B. (2007). Anti-inflammatory effects of compounds alpha-humulene and (−)-trans-caryophyllene isolated from the essential oil of *Cordia verbenacea*. Eur. J. Pharmacol..

[14] Zhang Q.Q., Lin L.G., Ye W.C. (2018). Techniques for extraction and isolation of natural products: A comprehensive review. Chin. Med..

[15] Machado B.A., de Abreu Barreto G., Costa A.S., Costa S.S., Silva R.P., da Silva D.F., Brandão H.N., da Rocha J.L., Nunes S.B., Umsza-Guez M.A. (2015). Determination of parameters for the supercritical extraction of antioxidant compounds from green propolis using carbon dioxide and ethanol as co-Solvent. PLoS ONE.

[16] Fischer B., Gevinski E.V., da Silva D.M., Júnior P.A.L., Bandiera V.J., Lohmann A.M., Rigo D., Duarte P.F., Franceschi E., Zandoná G.P. (2023). Extraction of hops pelletized (*Humulus lupulus*) with subcritical CO_2_ and hydrodistillation: Chemical composition identification, kinetic model, and evaluation of antioxidant and antimicrobial activity. Food Res. Int..

[17] Vichi S., Preedy V.R., Watson R.R. (2010). Extraction techniques for the analysis of virgin olive oil aroma. Olives and Olive Oil in Health and Disease Prevention.

[18] Olsovska J., Bostikova V., Dusek M., Jandovska V., Bogdanova K., Cermak P., Bostik P., Mikyska A., Kolar M. (2016). *Humulus lupulus* L. (hops)—A valuable source of compounds with bioactive effects for future therapies. Mil. Med. Sci. Lett..

[19] Radzali S.A., Markom M., Saleh N.M. (2022). Parameters effects and optimisation in supercritical fluid extraction of phenolic compounds form *Labisia pumila*. Separations.

[20] Sharif K.M., Rahman M.M., Azmir J., Mohamed A., Jahurul M.H.A., Sahena F., Zaidul I.S.M. (2014). Experimental design of supercritical fluid extraction—A review. J. Food Eng..

[21] Vafaei N., Rempel C.B., Scanlon M.G., Jones P.J.H., Eskin M.N.A. (2022). Application of supercritical fluid extraction (SFE) of tocopherols and carotenoids (hydrophobic antioxidants) compared to non-SFE methods. App. Chem..

[22] Pfaf-Šovljanski I.I., Grujić O.S., Peruničić M.B., Cvetković I.M., Zeković Z.P. (2005). Supercritical carbon dioxide hop extraction. Acta Period. Technol..

[23] Zeković Z., Pfaf-Šovljanski I., Grujić O. (2007). Supercritical fluid extraction of hops. J. Serb. Chem. Soc..

[24] Nagybákay N.E., Syrpas M., Vilimaitė V., Tamkutė L., Pukalskas A., Venskutonis P.R., Kitrytė V. (2021). Optimized supercritical CO_2_ extraction enhances the recovery of valuable lipophilic antioxidants and other consitutents from dual-purpose hop (*Humulus lupulus* L.) variety Ella. Antioxidants.

[25] Kupski S.C., Klein E.J., da Silva E.A., Palú F., Guirardllo R., Vieira M.G.A. (2017). Mathematical modeling of supercritical CO_2_ extraction of hops (*Humulus lupulus* L.). J. Supercrit. Fluid..

[26] Rój E., Tadić V.M., Mišić D., Žižović I., Arsić I., Dobrzyńska-Inger A., Kostrzewa D. (2015). Supercritical carbon dioxide hops extracts with antimicrobial properties. Open Chem..

[27] Del Valle J.M., Rivera O., Teuber O., Palma M.T. (2003). Supercritical CO_2_ extraction of Chilean hop (*Humulus lupulus*) ecotypes. J. Sci. Food Agric..

[28] He G., Xiong H., Chen Q., Ruan H., Wang Z., Traoré L. (2005). Optimization of conditions for supercritical fluid extraction of flavonoids from hops (*Humulus lupulus* L.). J. Zhejiang Univ.-Sci. B.

[29] Dzingelevičius N., Maruška A., Ragažinskienė O., Obelevičius K. (2011). Optimization of hop essential oil extraction by means of supercritical CO_2_. Biologija.

[30] Bizaj K., Škerget M., Košir I., Knez Ž. (2021). Sub- and supercritical extraction of Slovenian hops (*Humulus lupulus* L.) Aurora variety using different solvents. Plants.

[31] Schoss K., Kočevar G.N., Dolenc K.J., Anžlovar S. (2022). Supercritical CO_2_ plant extracts show antifungal activities against crop-borne fungi. Molecules.

[32] Du T., Shupe T.F., Hse C.Y. (2011). Antifungal activities of three supercritical fluid extracted cedar oils. Holzforschung.

[33] Tyśkiewicz K., Tyśkiewicz R., Konkol M., Rój E., Jaroszuk-Ściseł J., Skalicka-Woźniak K. (2019). Antifungal properties of *Fucus vesiculosus* L. supercritical fluid extract against *Fusarium culmorum* and *Fusarium oxysporum*. Molecules.

[34] Bai X., Cheng J., Liang W., Ma L., Liu Y., Shi G., Wang Y., Zhu E., Sambath S. (2012). Antifungal activity of extracts by supercritical carbon dioxide extraction from roots of *Stellera chamaejasme* L. and analysis of their constituents using GC-MS. Information Technology and Agricultural Engineering.

[35] Alonso-Esteban J.I., Pinela J., Barros L., Ćirić A., Soković M., Calhelha R.C., Torija-Isasa E., de Cortes Sánchez-Mata M., Ferreira I.C.F.R. (2019). Phenolic composition and antioxidant, antimicrobial and cytotoxic properties of hop (*Humulus lupulus* L.) seeds. Ind. Crops Prod..

[36] Nionelli L., Pontonio E., Gobbetti M., Rizzello C.G. (2018). Use of hop extract as antifungal ingredient for bread making and selection of autochthonous resistant starters for sourdough fermentation. Int. J. Food Microbiol..

[37] Bartmańska A., Wałecka-Zacharska E., Tronina T., Popłoński J., Sordon S., Brzezowska E., Bania J., Huszcza E. (2018). Antimicrobial properties of spent hops extracts, flavonoids isolated therefrom, and their derivatives. Molecules.

[38] Avalos J., Carmen Limón M. (2015). Biological Roles of Fungal Carotenoids. Curr. Genet..

[39] Naz T., Ullah S., Nazir Y., Li S., Iqbal B., Liu Q., Mohamed H., Song Y. (2023). Industrially Important Fungal Carotenoids: Advancements in Biotechnological Production and Extraction. J. Fungi..

[40] Mezghanni H., Khedler S.B., Tousni S., Zouari N. (2012). Medium optimization of antifungal activity production by Bacillus amyloliquefaciens using statistical experiment design. Prep. Biochem. Biotech..

[41] Mares Castro A., Estrada Monje A., Saldívar Campos A.I., Zaragoza Estrada A. (2023). Optimization of the Antifungal Property in a Composite of Polyurethane and Silver Nanoparticles against the *Trichophyton rubrum* Fungus. Appl. Sci..

[42] El-Housseiny G.S., Shams G., Ghobashi Z., Mamdouh R., Almaqsod I., Saleh S. (2021). Optimization of antifungal activity by Bacillus subtilis isolate CCASU 2021-4 using response surface methodology. Arch. Pharm. Sci. Ain Shams Univ..

[43] Gharbani P., Jam N., Doshmanfekan H., Mehrizad A. (2023). Optimization of synergic antibacterial activity of *Punica granatum* L. and Areca nut (P.G.L.A.N) extracts through response surface methodology. Sci. Rep..

[44] Chen C., Wan C., Peng X., Chen Y., Chen M., Chen J. (2015). Optimization of Antifungal Extracts from *Ficus hirta* Fruits Using Response Surface Methodology and Antifungal Activity Tests. Molecules.

[45] Pereira P., Bernardo-Gil M.G., Cebola M.J., Mauricio E., Romano A. (2013). Supercritical fluid extracts with antioxidant and antimicrobial activities from myrtle (*Myrtus communis* L.) leaves. Response surface optimization. J. Supercrit. Fluid..

[46] Lichtenthaler H. (1987). Determination of total carotenoids and chlorophylls a and b of leaf in different solvents. Methods Enzymol..

[47] Dere Ş., Güneş T., Sivaci R. (1998). Spectrophotometric determination of chlorophyll—A, B and total carotenoid contents of some algae species using different solvents. Tr. J. Bot..

